# Prevalence of Malnutrition in a National Sample of Older Adults Residing in Community or Residential Care: NHATS 2017

**DOI:** 10.1093/geroni/igaa057.2874

**Published:** 2020-12-16

**Authors:** Rose Ann DiMaria-Ghalili, Janeway Granche, Martha Coates, Zachary Hathaway, Justine Sefcik

**Affiliations:** Drexel University, Philadelphia, Pennsylvania, United States

## Abstract

The national prevalence of malnutrition in older adults living in the community and residential care (non-nursing home) is not known. We determined the prevalence of malnutrition (Mini-Nutritional Assessment) in a representative sample (N=4472) living in the community (95%) or residential care (5%), and examined known nutrition risk factors (inflammation [hsCRP, IL-6], socio-demographic variables). The majority (68%) were nourished, 26% were at risk, and 6% were malnourished. Those living in residential care vs community were more likely to be malnourished (12% vs 5%, respectively p<.01). Compared to nourished group, those with malnutrition were more likely to have hsCRP greater than median (1.36) (OR = 1.45 [95% CI 1.01-1.92]) and those at nutritional risk were more likely to have IL-6 greater than median (4.22) (OR=1.34 [95% CI 1.09-1.63]). Malnourished older adults were more likely to be older, female, live alone, report worse self-reported health, and use Meals on Wheels (p <.05).

